# Proportion of adults achieving sufficient physical activity increases in South Australia, 1998 – 2010

**DOI:** 10.1186/1478-7954-11-23

**Published:** 2013-12-03

**Authors:** Katherine Reta Devonshire-Gill, Kevin Ian Norton

**Affiliations:** 1 School of Health Sciences, University of South Australia, PO Box 2471, Adelaide, SA 5001, Australia

**Keywords:** Physical activity survey, Active Australia Survey, Insufficiently active

## Abstract

**Background:**

The South Australian Department of Health has administered the *Active Australia Survey* triennially since 1998 to assess physical activity levels in the South Australian adult population. Survey findings may reflect the impact of health messages on the population and provide evidence to inform public health policy.

This study analyzed the data from the South Australian *Active Australia Surveys* from 1998 to 2010 to quantify trends in the physical activity patterns of the total population and groups within the population over this period.

**Methods:**

The study used a retrospective analysis of the SA Health *Active Australia Surveys* of 1998, 2001, 2004, 2007, and 2010. Data were age-adjusted to the Australian 2001 Population Census. Proportions reporting sufficient physical activity for health benefits (≥ 150 min/week) were calculated by year of survey, and according to the following variables: gender, age group, area, educational level, income bracket, and body mass index (BMI). Population proportions statistics were used to determine the significance of the changes in levels of physical activity between 1998 and 2010.

**Results:**

The main finding was a significant increase in the overall proportion of the population reporting sufficient physical activity. However, the changes were not uniform across categories. Differences in proportions reporting sufficient physical activity appear to have narrowed within the categories of gender and BMI but have widened within the categories of educational level and income bracket. Changes were relatively consistent across age and geographic location.

**Conclusions:**

The results suggest that government programs to increase engagement in regular physical activity have been effective in some groups within the total population. They provide strong evidence to support the need for better ways of increasing physical activity levels and thus improving health outcomes for disadvantaged groups in the South Australian population.

## Background

The structure of the human body reflects its functional capacity for movement [1]. However, technological advances have made possible a lifestyle practically devoid of physical activity (PA) [2]. The negative consequences of this are exacerbated by the fact that not only are humans genetically predisposed to minimise energy expenditure, we are also hard-wired to store energy when food is abundant [3].

In the face of these social changes and pressures a plethora of studies has confirmed a graded, nonlinear relationship between PA levels and health outcomes. The greatest benefits are evident at the lower end of the scale when those who are sedentary or have low PA levels increase their activity [4,5]. The relationship between PA levels and mortality risk also follows the same pattern [6,7].

On this basis it is widely promoted that 30 minutes of moderate, or equivalent, physical activity a day is sufficient to result in substantial health benefits [8,9]. Living outside these guidelines increases risk factors for numerous chronic conditions such as cardiovascular disease, diabetes mellitus, colon and breast cancer, osteoporosis, and depression [6,10].

It is estimated that chronic disease accounts for approximately 80% of the burden of disease in Australia [11,12] and has rapidly risen throughout much of the world [13]. Physical inactivity has been estimated to account for 6.6% of the total disease burden in Australia [12], and in the state of South Australia (SA) approximately one-third of the entire budget is spent on health [14]. A study modeling the net health care costs associated with nonparticipation in PA in Australia estimated that the health care costs directly attributable to physical inactivity for the year 2006–2007 were almost $AUD1.5 billion [15].

National and state government departments recognize the contribution of PA to good health and the social and economic benefits of increasing PA levels in the population. Hundreds of initiatives have been developed to increase the proportion of the population engaging in PA [16,17]. In SA, like most states, this has translated into significant effort across the whole of government to ensure every department considers the impact of development on PA opportunities and health outcomes [18]. The measurement of population PA levels facilitates the evaluation of intervention programs and provides evidence to direct public health policy [18].

Accurately measuring PA patterns has been problematic, and consequently there is a range of methodologies used throughout the world [19-21]. However, the *Active Australia Survey* (AAS) was developed in 1997 to standardize the assessment of levels of nonoccupational PA in the Australian adult population [22]. This specific instrument has been used in over 40 state and national surveys since its inception, which permits assessment of longitudinal trends in PA patterns. In 1999, the Australian government established the National PA Guidelines to inform the public of the minimum amount of PA necessary to confer health benefits, essentially mirroring those published internationally [23]. The ‘cut-off’ level required for health benefits was set at 150 minutes of moderate, or equivalent, PA each week. These guidelines have been used consistently across Australia to monitor PA patterns of adults.

In 1998, the SA Department of Health first used the AAS in a random sample of South Australian adults to determine their PA patterns. The same survey has been conducted every three years. Although summary reports from the individual surveys have been published, data have not been amalgamated to determine the significance of trends across the series. This study combined data from the five SA PA surveys conducted between 1998 and 2010 to quantify PA trends and the relationships with a range of socio-demographic indices.

## Methods

This project was a retrospective analysis of the nonidentifiable AAS data contained within the SA Physical Activity Surveys of 1998, 2001, 2004, 2007, and 2010 [24-28]. For each survey the sample was selected using a standardized procedure [22]. All households in SA with a landline telephone connection and listed in the Electronic White Pages were eligible. Telephone numbers were selected using random direct dialing. Within each household, the person who most recently had their birthday and was at least 18 years of age was selected for the interview. There was no replacement for noncontactable persons, however there was a ring-back system used at least 10 times in order to interview the correct person. Exclusion criteria included respondents under the age of 18 and those who refused to give their age. Sample sizes (and response rates) were: 3059 (65.7%), 3995 (75.2%), 2999 (69.1%), 3065 (64.4%), and 3078 (65.0%) across the 1998 to 2010 surveys, respectively.

The CATI III (Computer Assisted Telephone Interview) system was used to interview participants. The system ensured correct sequencing of questions with automatic rotation of response categories to minimize bias. Data from the interviewer’s screen were immediately entered into the computer database.

Following ethics approval from the University’s Research Ethics Committee, a formal request to access the relevant PA databases was made to the data custodians, the Population Research and Outcome Studies (PROS), SA Health. On receipt of the databases the variable descriptions were closely scrutinized to ensure categories were consistent across surveys. The databases were transferred and amalgamated into a mega database file (Statview for Windows, version 5.0.1).

The independent variables, as defined in the South Australian PA surveys [24-28] were: gender, age, area, educational level, income bracket, and body mass index (BMI). The dependent variables were: number of times walking; number of minutes walking; number of times moderate PA; number of minutes moderate PA; number of times vigorous PA; and number of minutes vigorous PA. In cases where PA data were missing, a conservative approach was adopted, whereby 0 minutes was assumed. This was consistently applied across all surveys and represented 1.5% of all responses.

The Australian government has created two definitions to establish the sufficient level of physical activity (SPA) to confer health benefits: Definition 1 (D1) is 150 minutes total of walking, moderate or vigorous PA per week, with vigorous activity weighted by a factor of two. Definition 2 (D2) is 150 minutes total of walking, moderate or vigorous PA accrued over at least five sessions per week, with vigorous activity weighted by a factor of two [29]. Each subject on the mega database was coded to indicate if they achieved SPA according to each of the two definitions above. To avoid over-reporting, the data were truncated to a final maximum score of 3000 min/wk. This cut-off point accommodated the time spent in vigorous physical activity by professional athletes [30]. Scores for individuals reporting more than 3000 min were allocated a score of 3000 min. This was 0.025% of all cases.

### Statistical analyses

For each survey year the data were adjusted by age to the Australian 2001 Population Census allowing the final estimated proportions of adults SPA to be compared. For each survey year, the proportions of sufficiently active individuals in each of the following categories were calculated using raw data: gender, age, area, educational level, income bracket, and BMI category. Inferential statistics comparing two population proportions (z-scores) were used to test the hypothesis that the proportion of adults in the SA population achieving SPA had increased between the 1998 and 2010 surveys. The level of significance was set at α <0.10. Despite multiple pairwise comparisons between variables collected in the 1998 and 2010 surveys, Bonferroni corrections were not applied to the test results. Rather, the original probability levels for the comparisons are provided and these can be placed into context of the overall trends and patterns of changes among the variables measured [31].

## Results

The proportions of physically active survey participants within the various categories are shown in Tables 1 and 2. These tables present the summary data for all five surveys. Table 1 uses D1 to calculate the proportions, whereas Table 2 uses D2. Pairwise comparisons between the 1998 versus 2010 surveys have been calculated and the probability levels are indicated.

**Table 1:** The proportions of each category achieving SPA in the five SA PA surveys using D1 (total weekly PA ≥150 min)

**SA PA DATA 1998 – 2010 D1**							
**CATEGORY**	**D1**					***n***	***p***
	**1998**	**2001**	**2004**	**2007**	**2010**	**98/10**	**98-10**
**TOTAL SAMPLE**							
Non-standardized	**52.2**	**49.6**	**51.0**	**53.4**	**56.7**	3059/3078	<0.001
Age-standardized*	**53.4**	**52.0**	**53.9**	**57.1**	**61.5**	3059/3078	<0.001
**GENDER**							
Male	**56.6**	**55.1**	**54.5**	**56.0**	**59.8**	1262/1280	0.095
Female	**49.2**	**45.4**	**48.2**	**51.7**	**54.4**	1797/1798	<0.002
**AGE GROUP** [yr]							
18-24	**69.1**	**66.7**	**72.9**	**69.2**	**77.1**	291/144	0.081
25-34	**59.3**	**56.8**	**57.2**	**62.0**	**68.3**	501/249	0.017
35-44	**54.0**	**54.3**	**55.1**	**58.8**	**62.8**	676/471	0.003
45-54	**49.3**	**45.3**	**50.0**	**58.3**	**59.3**	560/612	0.001
55-64	**53.4**	**52.5**	**48.6**	**56.7**	**55.6**	401/626	0.485
65-74	**45.7**	**42.7**	**50.1**	**47.1**	**53.2**	370/543	0.025
75+	**29.2**	**35.1**	**33.5**	**30.0**	**38.8**	260/433	0.011
**AREA**							
Metropolitan	**53.5**	**51.2**	**52.5**	**53.9**	**58.0**	2301/2258	0.002
SA country [rural]	**48.4**	**45.7**	**47.3**	**52.5**	**53.2**	758/820	0.059
**EDUCATIONAL LEVEL**							
Year 12	**48.4**	**45.7**	**44.7**	**46.4**	**49.9**	1732/1402	0.390
Certificate/diploma	**55.1**	**53.2**	**53.0**	**57.5**	**57.5**	942/1043	0.275
University degree	**62.6**	**57.5**	**67.3**	**68.1**	**70.8**	385/613	<0.001
**INCOME BRACKET** [AU$]							
<$20 000	**46.7**	**41.3**	**40.9**	**39.5**	**38.6**	1014/513	0.002*
$20 000 - $40 000	**53.2**	**51.1**	**50.8**	**50.4**	**55.7**	679/549	0.369
$40 001 - $60 000	**56.2**	**51.8**	**54.0**	**55.8**	**58.0**	502/398	0.575
$60 000+	**60.8**	**57.2**	**59.2**	**65.6**	**66.5**	444/1103	0.033
**BMI** [kg/m^2^]							
<18.5	**50.0**	**53.2**	**37.9**	**48.9**	**45.3**	70/53	0.604
18.5 - 24.9	**57.3**	**55.7**	**57.4**	**60.2**	**60.6**	1427/1102	0.095
25.0 - 29.9	**52.4**	**48.4**	**50.2**	**53.5**	**59.5**	967/1022	0.002
30+	**41.0**	**40.6**	**42.9**	**44.6**	**51.2**	417/648	0.001

The sample sizes and significance of the changes in proportions (from 1998 to 2010) are shown. *indicates a significant decrease in proportion.

**Table 2:** The proportions of each category achieving SPA in the five SA PA surveys using D2 (total weekly PA ≥150 min and session number ≥5)

**SA PA DATA 1998 – 2010 D2**							
**CATEGORY**	**D2**					***n***	***p***
	**1998**	**2001**	**2004**	**2007**	**2010**	**98/10**	**10-98**
**TOTAL SAMPLE**							
Non-standardized	**40.3**	**38.3**	**40.2**	**43.4**	**43.5**	3059/3051	0.012
Age-standardized*	**41.4**	**40.2**	**42.6**	**46.5**	**47.8**	3059/3051	<0.001
**GENDER**							
Male	**44.3**	**43.0**	**42.5**	**46.5**	**46.2**	1262/1268	0.332
Female	**37.6**	**34.8**	**38.4**	**41.4**	**41.6**	1797/1783	0.014
**AGE GROUP** [yr]							
18-24	**58.1**	**53.1**	**57.4**	**56.2**	**62.1**	291/140	0.421
25-34	**45.3**	**44.1**	**45.8**	**50.2**	**54.1**	501/246	0.024
35-44	**40.5**	**42.8**	**44.6**	**47.8**	**47.9**	676/468	0.014
45-54	**37.7**	**34.2**	**39.8**	**48.5**	**47.1**	560/607	0.001
55-64	**40.4**	**40.7**	**40.1**	**45.5**	**41.0**	401/622	0.849
65-74	**34.9**	**32.1**	**36.1**	**39.0**	**41.7**	370/535	0.039
75+	**23.8**	**26.7**	**24.1**	**23.3**	**27.5**	260/433	0.291
**AREA**							
Metropolitan	**41.2**	**39.5**	**41.7**	**43.6**	**44.4**	2301/2237	0.032
SA country [rural]	**37.6**	**35.4**	**36.5**	**43.1**	**41.0**	758/814	0.164
**EDUCATIONAL LEVEL**							
Year 12	**35.9**	**35.7**	**34.3**	**37.3**	**38.1**	1732/1390	0.217
Certificate/diploma	**43.8**	**38.9**	**41.9**	**47.9**	**43.0**	942/1035	0.704
University degree	**51.7**	**47.1**	**56.1**	**55.1**	**56.8**	385/606	0.118
**INCOME BRACKET** [AU$]							
<$20 000	**36.4**	**31.2**	**30.1**	**31.9**	**29.2**	1014/510	0.005*
$20 000 - $40 000	**38.6**	**41.1**	**40.3**	**40.8**	**43.6**	679/543	0.074
$40 001 - $60 000	**44.0**	**42.0**	**43.0**	**45.6**	**44.2**	502/396	0.960
$60 000+	**48.6**	**43.9**	**48.0**	**53.4**	**52.2**	444/1093	0.213
**BMI** [kg/m^2^]							
<18.5	**41.4**	**40.3**	**31.8**	**44.9**	**37.7**	70/53	0.679
18.5 - 24.9	**45.1**	**45.3**	**44.2**	**48.8**	**47.7**	1427/1087	0.208
25.0 - 29.9	**39.1**	**35.8**	**39.8**	**43.9**	**46.3**	967/1016	0.001
30+	**30.7**	**29.0**	**35.1**	**36.1**	**36.5**	417/646	0.050

The sample sizes and significance of the changes in proportions (from 1998 to 2010) are shown. *indicates a significant decrease in proportion.

The overall trends for the 1998 to 2010 period were relatively consistent and positive despite a decrease in the proportion of SPA from 1998 to 2001. There was a significant increase in the proportions of the SA adult population achieving SPA from 1998 to 2010 under either definition. Using the age-standardized data the increases in SPA were equivalent to 8.1% and 6.4% for D1 and D2, respectively.

The tables show a greater proportion of males achieved SPA at both time points in comparison to females. However, females increased to a greater extent in the period from 1998 to 2010 and appear to be closing the gap. Both D1 and D2 show the SPA gap narrowed by approximately two percentage points across the period in review.

The results reveal an inverse relationship between age and proportions achieving SPA. In both surveys the youngest group achieved over twice the level of that achieved by the oldest group. Importantly, the positive changes over the 12-year period were consistent across the age groups (range = +7.5 to 10%) with the exception of the 55–64 year group (+2.2%).

A greater proportion of survey respondents living in the Adelaide metropolitan area reported SPA than those living in rural areas. The difference was consistent across the period of the surveys, approximately 5% by D1 and 3.5% by D2. Both categories showed similar increases in SPA from 1998 to 2010; 4.5% for metropolitan and 4.8% for rural [D1], and by 3.2% for metropolitan and 3.4% for rural [D2].

The surveys consistently reveal a pattern of higher levels of education linked with higher proportions of SPA. The gap in SPA between the lowest and highest educational levels increased from 14.2% in 1998 to 20.9% in 2010 [D1]. Although there was an upward trend from 1998 to 2010 in proportions of SPA at each educational level, the only change to reach the significance level set was in the most highly educated category [university degree], with an increase of 8.2% [D1].

There was a strong association between proportions achieving SPA and level of household income, with a greater proportion of higher-income populations reporting SPA than lower-income groups. Using D1, the only category to achieve a significant increase in SPA was the highest income group, with a change of 5.7%. By contrast, the lowest income group showed a significant decrease in SPA of 8.1% (7.6% using D2). The second-lowest income bracket showed an increase in SPA of 5% using D2.

Tables 1 and 2 show a clear gradient in SPA with increasing BMI - the higher the BMI, the lower the proportion reaching SPA. In 1998 there was a 4.9% difference between normal and overweight and a 16.3% difference between normal and obese categories [D1]. This general trend continued across surveys. However, the gap in SPA according to BMI lessened markedly. By 2010 these differences in the proportions had reduced to 1.1% and 9.4%, respectively.

## Discussion

This paper amalgamated five consecutive PA surveys conducted in SA to assess changes in proportions of the adult population achieving SPA across the period 1998 to 2010. The main findings were that overall proportions reporting SPA increased and that these changes were relatively consistent across various age groups. However, while the gap in proportions achieving SPA appears to have narrowed in the categories of Gender and BMI, it has widened in the categories of educational level and income bracket.

The importance of these modest increases cannot be understated. For example, if the results of these surveys were to reflect the PA behavior patterns of the total Australian population, it suggests that in 2010 there were over a million more adults undertaking SPA to gain health benefits than in 1998. Is this a reasonable extrapolation? Trend data have become available from time to time in other states where a total of at least 40 repeat surveys using the same PA instrument have been conducted since 1997. Although these datasets have not been amalgamated, which misses a foremost opportunity to examine national trend data, they still provide an insight into patterns within individual states. For example, significant increases in SPA among adults have been reported for New South Wales [32], Victoria [33,34], Queensland [35] and Western Australia [36] based on their state-based surveys. In Qld and NSW, the increases were almost 9%. Vic showed a 5.4% increase, but had the highest proportions of SPA of the mainland states. On the other hand, WA revealed a different pattern, with a decrease in SPA of 7.3% from 2003 to 2006, followed by an increase of 9.6% from 2006 to 2010. Therefore, there is reasonable evidence for small but significant incremental steps toward higher levels of leisure-time PA among adult Australians.

This increase across Australia, and within SA specifically, may be due to any number of factors, and it is impossible to disentangle what has been significant in altering PA behaviors. For example, innumerable health initiatives have been introduced during this period including local, regional, state, and national efforts costing billions of dollars. Some of the major national initiatives that focus on ways of increasing PA include the *National Physical Activity Guidelines*[8], *Preventable Chronic Diseases Strategy*[37], *National Healthy Ageing Strategy*[38] and the *Australian Better Health Initiative*[39] that incorporated aspects such as the *Measure-up* campaign. There have also been nationwide *Get Moving*[40], *Active Australia*, *Healthy Communities*[41] and *Swap it-don’t’ stop it* campaigns [42] focusing on strategies to increase public awareness of the benefits of regular PA and to provide greater opportunities for people to be physically active. There are even more examples within each state that have promoted healthy behaviors including PA [43-47].

Large-scale population surveys studies in Australia and overseas have consistently shown that males report higher levels of leisure time PA than females [29,48,49]. The results of this study support these findings. However, over the period 1998 to 2010, although both genders reported increased levels of SPA, the absolute change was greater in the female population, especially by D2. Although the gym and recreation center attendance represents only 14% of the adult population it has grown substantially in recent years and is particularly popular among women. This suggests that many women are tending to do more exercise ‘little and often’, perhaps taking advantage of specifically designed 30 minute programs and ‘women only’ gyms [50].

The findings of the current study indicate that generally no particular age group has been impacted more or less than any other to increase PA levels, and the entire population curve has shifted slightly upwards. For example, there is an approximate two-fold difference between the youngest and oldest groups, and this has remained relatively stable. It is clearly a biological phenomenon that PA levels decrease as we age. Decreases of about 50% across the adult age range are consistently seen throughout most of the animal kingdom [51]. However, it is still of concern that the demographic shift towards an aging population, in combination with the broad and persistently low SPA levels and the associated increasing prevalence of chronic conditions, will present even greater challenges in the future. Several specifically targeted promotion campaigns have been launched for the elderly. The *Rusty* (tin man) promotion in 1999 and the *National Healthy Ageing Strategy* are two examples [29,38]. They specifically targeted older age groups and may have contributed to the increase of about 9% in the proportion of over 75 year olds engaging in SPA.

The results suggest that where one lives may impact on PA behaviors. Across the period of the surveys, a greater proportion of respondents living in the Adelaide metropolitan area consistently reported SPA than their rural counterparts by about five percentage points. Health inequalities between metropolitan and rural Australia have been widely reported in the literature [52]. Environmental factors, physical access to services, and socio-economic status (SES), cited as reasons for the health differential, might explain the disparity in SPA levels according to location [53]. Other reasons may involve higher levels of physical labor of many occupations in rural communities that are not captured in the AAS. However, this possibility is likely to be diminishing as technologies infiltrate more [54] and may be reflected in rural BMI levels that are higher across all percentile bands in the current mega dataset (21.1 vs 20.6; 23.4 vs 22.7; 26.1 vs 25.3; 29.4 vs 28.6; and 33.2 vs 32.4 for the 10^th^, 25^th^, 50^th^, 75^th^, and 90^th^ percentiles, respectively).

Findings from studies across Australia and overseas repeatedly show a relationship between SES and PA behavior patterns [55-57]. Two aspects of SES were examined in this study, highest educational level attained and annual household income. Both categories followed a similar pattern from 1998 to 2010, with higher educational levels and greater income being linked with higher proportions of SPA. The data show the gap between the disadvantaged and the advantaged populations has increased. For example, D1 shows that elevated PA levels within both the highest education and income categories have contributed to the widening gaps. Importantly, this has been made worse by the marked decrease in the SPA patterns of the lowest income group. It is often assumed higher educational levels may facilitate comprehension of health messages which then inform PA behaviors. However, approximately 95% of adult Australians indicate they know PA is good for their health and this proportion did not vary among education categories [29]. Nonetheless, educational attainment levels have risen over time. In the 1998 PA survey, 56% were in the lowest education category; this fell to 45% in 2010. In contrast, those at the highest end of the spectrum rose from 13% to 20% over the same time span.

When analyzing longitudinal data with respect to income, it is important to be cognizant of the increase in economic development and its effect on inflation. The poverty line (updated using the Consumer Price Index) has risen from $440/week in 1998–99 to $832/week in 2010–2011 [58]. Clearly the household disposable income of the overall population is increasing, but the income brackets have remained constant in each survey. Therefore, inflation has resulted in a greater proportion of the respondents being in the uppermost income bracket, progressively leaving only the most marginalized households in the lowest category and exacerbating the decrease in SPA levels in this group. Notwithstanding, encouraging and supporting the most disadvantaged groups to become more physically active has the greatest potential to reduce health inequalities and should remain a priority area.

The positive changes in self-reported SPA in the overweight and obese populations might be in part a response to government public education programs to increase people’s awareness of the health dangers associated with excessive body weight. However, they might also be a reflection of the change in rates of overweight and obesity in the total population. Based on measured BMI, 63.4% of the Australian population were overweight or obese in 2011–2012 [59]. The proportion overweight remained relatively stable from 1998 to 2010, but the proportion obese rose by five percentage points as the population BMI frequency distribution shifted to the right [60]. The PA data show that higher proportions of normal weight respondents report SPA than the overweight and obese groups. The higher proportions of SPA in the total population from 1998 to 2010 might indicate people are maintaining their exercise behaviors in spite of increasing their body mass over time.

## Limitations

There are a number of limitations involved in this series analysis. The samples were limited to listings in the Electronic White Pages, thereby excluding households without a landline or with unlisted numbers. Although the samples were randomized, participation in the survey was voluntary. However, the response rates were similar across the five surveys. Data are always subject to social desirability bias that would result in an overestimation of true proportions. Given the strong social media and awareness campaigns to promote PA, it is possible this has had some, unquantifiable influence on the trends identified. The AAS excludes certain domains of PA, notably occupational, housework, and gardening PA. Therefore, the data do not reflect total PA, only leisure-time activity. Self-report questionnaires inevitably fall prey to recall bias, and differences in interpretation of the questions create other sources of error. For example, PA intensity is judged subjectively and each person's response is colored by perceptions of their behavior and possibly fitness, and these may change over time.

## Conclusions

Population surveillance is an invaluable tool for gaining evidence to inform practices that improve the health of the community and its individuals. *The Active Australia Survey* is a standardized instrument for assessing population PA behavior.

An analysis of the data from the South Australian *Active Australia Surveys* from 1998 to 2010 revealed an upward trend in the PA patterns of the overall South Australian adult population over the period of review, but the improvements were not consistent across the categories. The most significant positive changes were seen in the categories of gender (females) and BMI (obese). A disturbing finding was an increase in the disparity between disadvantaged and advantaged populations, with a significantly lower proportion of respondents in the lowest income bracket reporting SPA in 2010 than in 1998.

The findings suggest that government programs to increase public awareness of the importance of regular PA may have impacted positively on some subpopulations within the total population, thus improving health outcomes in these groups. However, further efforts need to be directed toward ways of improving health outcomes for those most at risk.

This study is the first part of a larger study to determine to what extent the South Australian trends are reflected in the PA patterns of the adult population nationally.

## Competing interests

The authors declare that they have no competing interests.

## Authors’ contributions

KD-G carried out the data collation and analysis, and drafted the manuscript. KN contributed to the project design and to the manuscript. All authors read and approved the final manuscript.
